# Expanded Polyfunctional T Cell Response to Mycobacterial Antigens in TB Disease and Contraction Post-Treatment

**DOI:** 10.1371/journal.pone.0011237

**Published:** 2010-06-21

**Authors:** James M. Young, Ifedayo M. O. Adetifa, Martin O. C. Ota, Jayne S. Sutherland

**Affiliations:** Bacterial Diseases Programme, Medical Research Council Laboratories, Banjul, The Gambia; Institute of Infectious Diseases and Molecular Medicine, South Africa

## Abstract

**Background:**

T cells producing multiple factors have been shown to be required for protection from disease progression in HIV but we have recently shown this not to be the case in TB. Subjects with active disease had a greater proportion of polyfunctional cells responding to ESAT-6/CFP-10 stimulation than their infected but non-diseased household contacts (HHC). We therefore wanted to assess this profile in subjects who had successfully completed standard TB chemotherapy.

**Methods:**

We performed a cross-sectional study using PBMC from TB cases (pre- and post-treatment) and HHC. Samples were stimulated overnight with TB antigens (ESAT-6/CFP-10 and PPD) and their CD4+ and CD8+ T cells were assessed for production of CD107a, IFN-γ, IL-2 and TNF-α and the complexity of the responses was determined using SPICE and PESTLE software.

**Results and Conclusions:**

We found that an increase in complexity (i.e., production of more than 1 factor simultaneously) of the T cell profile was associated with TB disease and that this was significantly reduced following TB treatment. This implies that T cells are able to respond adequately to TB antigens with active disease (at least initially) but the ability of this response to protect the host from disease progression is hampered, presumably due to immune evasion strategies by the bacteria. These findings have implications for the development of new diagnostics and vaccine strategies.

## Introduction

Tuberculosis (TB) is a global health problem with 2 billion people infected with the causative agent *Mycobacterium tuberculosis* (MTb). Of these, 10 percent will progress to active TB disease resulting in almost 2 million deaths per year [1]. Due to the large discrepancy between the number of people infected and those who progress to active disease, there appears to be an adequate immune response in most people, at least initially. The requirements for a protective immune response are yet to be fully elucidated but include changes in the host immune system together with changes in the virulence and pathogenesis of the *Mycobacterium*.

MTb is an intracellular pathogen that infects macrophages and triggers a cascade of cell-mediated immune responses. IFN-γ producing CD4+ T cells provide the major effector response to TB but while IFN-γ is required for protection against disease progression in TB it is not sufficient on its own [2]. TNF-α has also shown to be protective as shown by the increased incidence of TB in patients given anti-TNF treatments for autoimmune diseases [3]. T cells that produce multiple factors simultaneously are termed polyfunctional T cells (PFT) and have been shown to provide protection against disease progression in HIV-1 infection [4] and also vaccine induced immunity [5]. Surprisingly, we have recently shown that this same profile of response to Tuberculosis (TB) antigens is higher in patients with active disease compared to latent infection following stimulation with ESAT-6/CFP-10 [6] suggesting this phenotype is not protective in the TB setting. Variable results have been seen in other studies [7], [8] dependent on the cytokines of interest, the antigenic stimuli, the age of the subjects and their genetic background. Not surprisingly, the differences seen in active disease depend on disease severity and the site of analysis: increased antigenic load in advanced pulmonary TB has been shown to correlate with decreased IFN-γ production in the blood, but increased production in the lungs [9]. Determination of the T cell cytokine profile at specific stages of infection, disease and recovery is critical for development of new diagnostics and vaccine strategies.

For a particular immune profile to be associated with TB disease, abrogation or reversal needs to be shown following standard treatment regimes for TB. As such, we compared the PFT cell profiles of TB cases before and after treatment to that seen in latently infected household contacts (HHC) following stimulation with MTb antigens. We found a significant increase in the proportion of both CD4+ and CD8+ T cells expressing CD107a, IFN-γ and TNF-α but not IL-2 in patients with active TB disease prior to treatment compared to post-treatment responses. Following successful TB treatment, the proportion of cytokine positive cells was reduced to levels equivalent to that seen in HHC. Furthermore, subjects with active TB disease had significantly higher levels of T cells producing 2 or more factors which were again reduced following treatment. These findings have implications for development of new diagnostics and vaccine strategies.

## Results

### Production of CD107a, IFN-γ, IL-2 or TNF-α by T cells following MTb antigen-specific T cells is increased in TB cases at recruitment but comparable to HHC post-treatment

**Figure 1:**
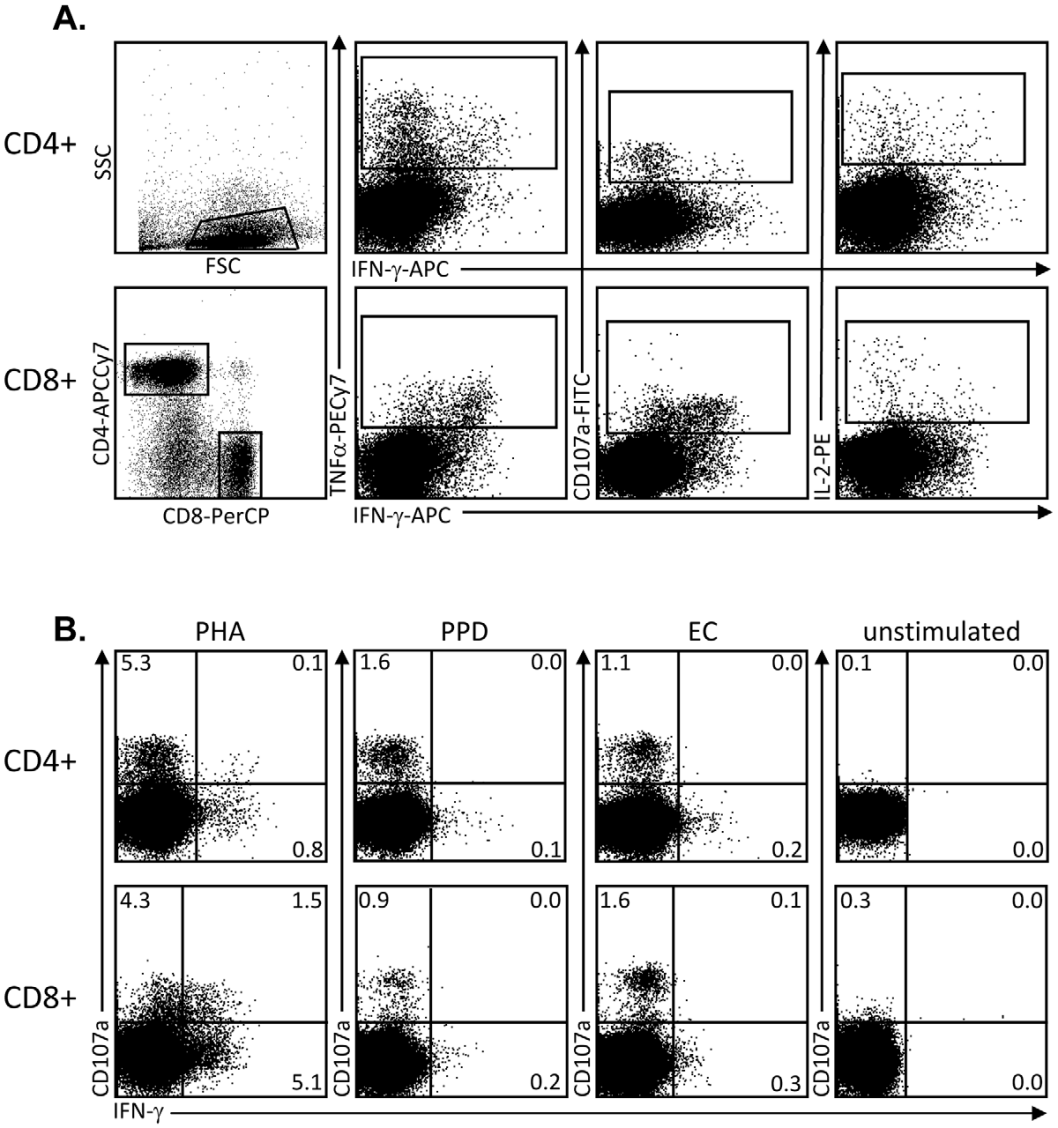
Representative flow cytometry profiles demonstrating the gating strategy used throughout the study. **A)** PBMC were stimulated overnight and cells were analysed by flow cytometry for expression of CD107a, TNF-α, IL-2 and IFN-γ. Following lymphocyte gating, CD4+ and CD8+ T cells were analysed separately for combinations of all 4 factors. B) Representative dot plots illustrating cytokine production from CD4+ and CD8+ T cells following PHA, PPD, EC and no stimulation.

We analysed production of total IFN-γ, IL-2, TNF-α and CD107a following overnight stimulation with ESAT-6/CFP-10 (EC), PPD-T and PHA as a positive control (Table 1 and Figure 1). Figure 1 illustrates the gating strategy used in this study with CD4+ and CD8+ T cells assessed separately for expression of CD107a, IFN-γ, IL-2 and TNF-α (Fig. 1A). CD4+ T cells generally had higher IFN-γ and TNF-α production than CD8+ T cells, particularly following PPD and EC stimulation while CD8+ T cells were more likely to have co-expression of CD107a with IFN-γ and/or TNF-α (Figures 1A and B).

**Table 1:** Proportion of CD4+ or CD8+ cells positive for any of the 4 factors (CD107a, IFN-γ, IL-2 or TNF-α) following overnight antigenic stimulation.

	%CD4			%CD8		
	Cases	Cases	Contacts	Cases	Cases	Contacts
Stimulation	pre-treatment	post-treatment	TST+	pre-treatment	post-treatment	TST+
PHA	10.5[6.6–13.4]	4.1[2.1–8.5]*	4.2[2.7–6.2]**	17.3[15–24.2]	6.4[1.6–11.9]***	5.4[3.5–7.4]***
PPD	3.7[2.2–6.3]	2.4[1.1–3.4]	2.8[0.7–4.2]	12[6.5–14.4]	2.1[1.8–3.7]**	2[0.7–4.5]**
EC	7.6[4]–[10]	2[1.2–3.4]**	2.3[0.7–4]**	12[5.5–13]	2.5[1.9–3.6]**	2.7[1.1–5.4]**

Data expressed as median [interquartile range (IQR)]-positive cells per subset.

* = p≤0.05;

** = p≤0.01,

*** =  p≤0.001 compared to TB cases pre-treatment.

The proportion of CD4+ or CD8+ cells positive for any one factor following PHA stimulation was significantly increased in TB cases before treatment compared to both HHC and cases following treatment (p<0.01 and p<0.05 respectively; Table 1) indicating that cells from TB cases prior to treatment are more responsive to general stimulation. PPD and ESAT-6/CFP-10 peptides (EC) were used to elicit a TB-specific response. TB cases had significantly higher levels of cytokine-positive cells than HHC within both the CD4+ and CD8+ T cell subsets following EC stimulation and the CD8+ subset following PPD stimulation (p<0.01 for all; Table 1). CD8+ levels were high (12% positive in TB cases following both PPD and EC stimulation) due to high expression of CD107a. The proportion of CD4+ and CD8+ cells positive for any one factor were significantly reduced to similar levels as HHC following successful TB treatment (Table 1).

**Figure 2:**
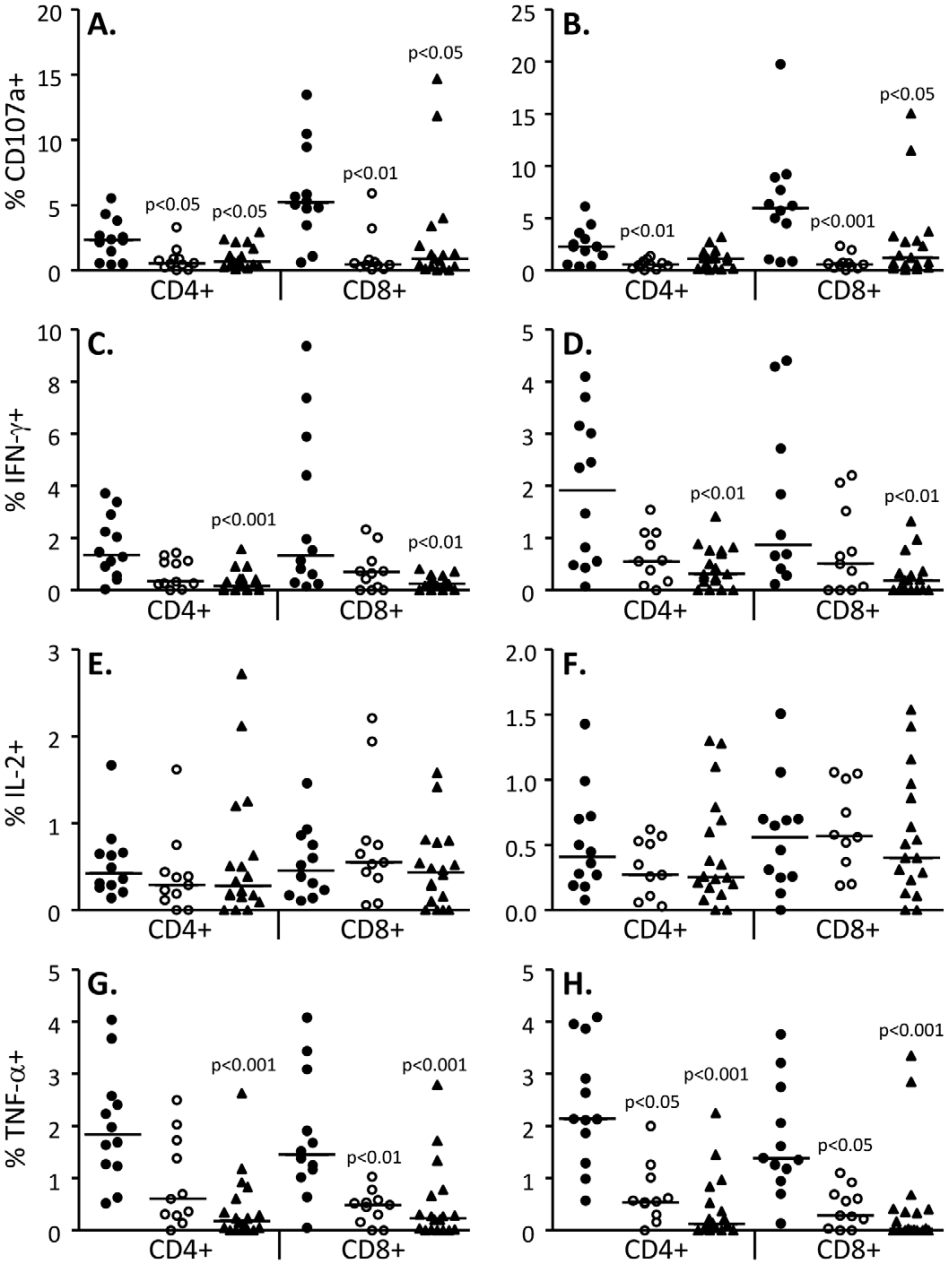
Increased CD107a, IFN-γ and TNF-α expression in TB cases pre-treatment and reduction post-treatment. Bar indicates median of TB cases pre-treatment (n = 12, filled circles), TB cases post-treatment (n = 10, open circles) and 17 HHC (triangles). Data were analysed using a Kruskal-Wallis ANOVA followed by Dunn's post-test comparison and p-values indicated.

All our results following analysis of 4 factors were consistent with our previous findings analysing 3-factors [5] with TB cases generally showing significantly more expression of CD107a, a known marker of degranulation and thus indicative of the cytotoxic potential of the cells [10]. They also had significantly higher production of most other cytokines than HHC in response to TB antigens (Figure 2). Importantly, following standard treatment for TB, these levels were similar to those seen in the TST+ (latently infected) household contacts (HHC). Due to its cytotoxic role, CD107a expression was higher in CD8+ compared to CD4+ cells and was significantly higher in TB cases compared to both post-treatment cases and HHC. We also found a significantly higher level of TNF-α production from both CD4+ and CD8+ T cells in TB cases pre-treatment compared to both TB cases post-treatment and HHC (Figure 2).

### Expanded polyfunctional T cell responses in TB cases and contraction following treatment

**Figure 3:**
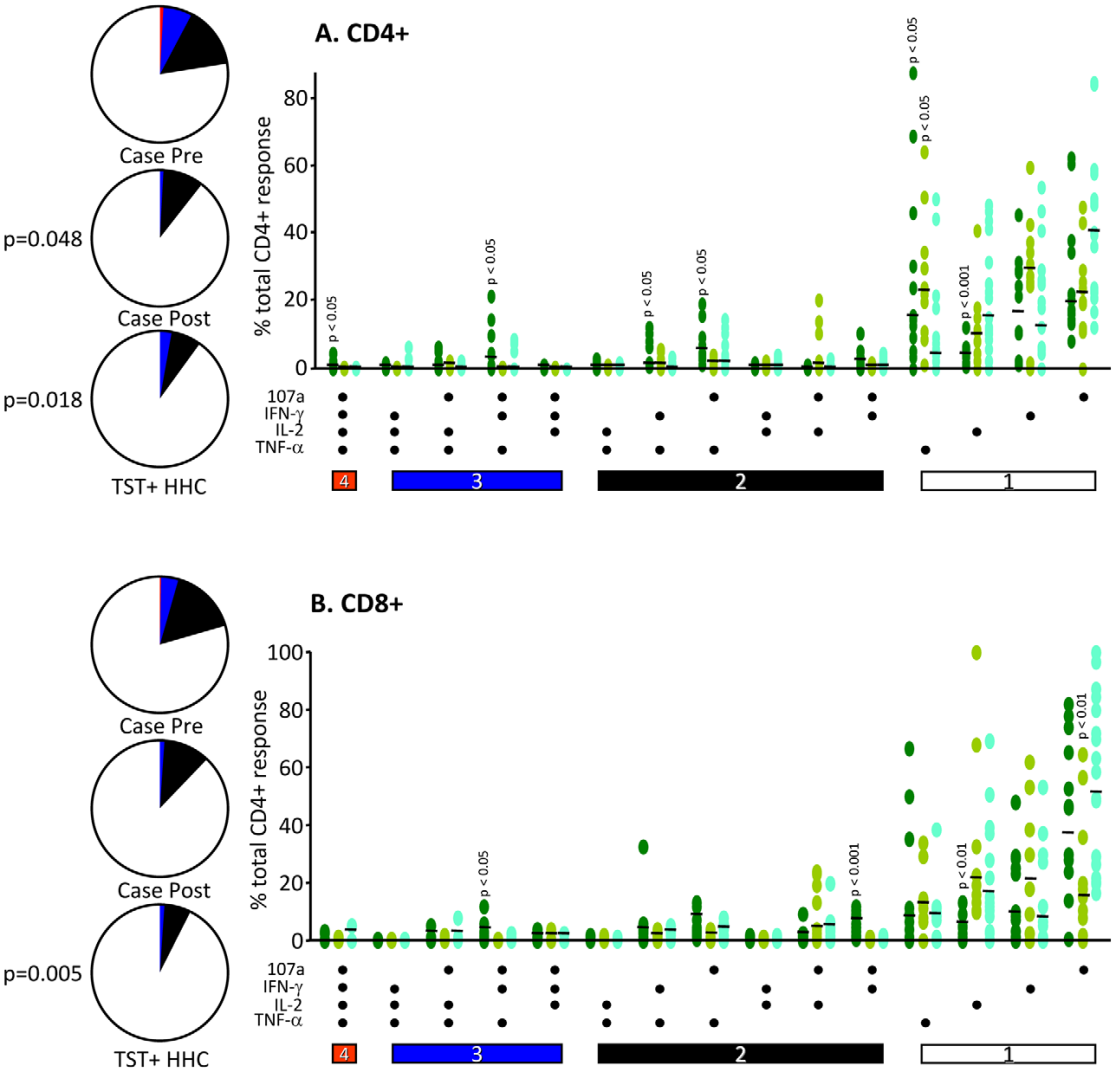
The functional profile of CD4+ and CD8+ T cells is increased in TB cases but reduced post-treatment. PBMC were stimulated overnight using ESAT-6/CFP-10 and CD4+ and CD8+ cells were analysed by flow cytometry for different combinations of IFN-γ, IL-2, TNF-α and CD107a. Responses are grouped and colour coded according to the number of factors: White  = 1 function, black  =  any 2 factors, blue  =  any 3 factors and red  =  all 4 factors. The pie charts summarise the results shown in the dot plots indicating the combination of cytokines as a percent of total responding cells for CD4+ (A) and CD8+ (B). Significant differences are indicated and were determined using a Kruskal-Wallis ANOVA followed by Dunn's post-test comparison. Pie chart segments were analysed using a permutation test and p-values indicated.

We next assessed the combination of CD107a, IFN-γ, IL-2 and TNF-α produced by both CD4+ and CD8+ T cells in TB cases pre and post-treatment compared to HHC following overnight stimulation with ESAT-6/CFP-10. Prior to treatment, TB cases had significantly higher levels of CD4+ T cells producing 2 or more factors simultaneously compared to TB cases following treatment and HHC (p = 0.048 and p = 0.018 respectively; Figure 3A, pie graphs). This was seen mainly for combinations involving TNF-α with significantly higher levels of CD4+107a+IFN-γ+IL-2+TNF-α+, together with CD4+107a+IFN-γ+TNF-α+, CD4+IFN-γ+TNF-α+ and CD4+107a+TNF-α+ cells (p<0.05 for all; Figure 3A). Interestingly, the proportion of CD4+ cells producing TNF-α alone was significantly higher in TB cases prior to treatment compared to HHC and this was not reduced following treatment (p<0.05 for both; Figure 3A). However TB cases pre-treatment had significantly lower CD4+ cells expressing IL-2 alone compared to cases post-treatment and to HHC (p<0.001; Figure 3A). CD8+ T cells from TB cases pre-treatment also showed significantly reduced levels of cells expressing IL-2 alone but no difference in expression of other single cytokines (p<0.01; Figure 3B). Interestingly, there was a significantly reduced proportion of CD8+ T cells expressing CD107a alone in TB cases post-treatment compared to pre-treatment cases and HHC (p<0.01; Figure 3B). Similar to the pattern seen for CD4+ T cells, CD8+ T cells from TB cases pre-treatment had a significantly higher proportion of cells producing more than 1 factor simultaneously compared to the HHC (p = 0.005; Figure 3B, pie graphs). This was reduced but not significantly in TB cases post-treatment. This significance was due mainly to an increase in CD8+CD107a+IFN-γ+TNF-α+ and CD8+CD107a+IFN-γ+ cells in the TB cases pre-treatment (p<0.05 and p<0.001 respectively; Figure 3B).

## Discussion

This study assessed the functional differences in T cell responses to Mycobacterial antigens depending on the infection status of the individual. We found that the complexity of the T cell response to TB antigenic stimulation was much higher in subjects with active TB disease than in latently infected household contacts and that this response was contracted following successful TB treatment. This indicates that the initial progression of TB disease is not due to impaired T cell function but most likely is determined by pathogen related factors which hamper the ability of the T cells to provide protective immune responses.

Production of multiple cytokines has been associated with protection from disease progression in HIV [4] but not TB [6]. Most studies assessing possible protective cytokines to MTb infection have measured only single cytokines, or multiple cytokines in the context of post-vaccination responses [7], [8], [11]. Furthermore, most information has been obtained using ELISPOT or ELISA which do not indicate the source of cytokine production. Given that both CD4+ and CD8+ T lymphocytes contribute to protection against TB [12], we assessed multiple cytokine production from both T cell subsets in response to MTb antigens using multi-colour flow cytometry. Clearly phenotypic analysis does not necessarily equate to actual cell function, but on the basis of these analyses, we show that TB cases have a more complex functional profile prior to treatment and this is reduced to HHC levels post-treatment. These findings implicate factors other than effector T cells in the initial progression to active TB disease. In more advanced TB disease T cell function has been shown to be reduced [13] indicating that biomarkers of disease progression need to be determined based on a correlation with bacterial load [14]. However, further studies should also focus on the antigen-specific response within the lung as sequestration can occur during acute disease resulting in a low response from peripheral blood cells compared to those from the lung [7], [9]. This has implications for TB diagnostics based on cytokine responses and supports the premise for biomarkers to reflect different stages of disease [14].

There are other potential contributing factors to this anomaly of the immune system: why an observed increase in T cell function in TB disease does not equate to protection from disease progression. These include an increase in T regulatory cells in active TB disease [15], changes in the ratio of Th1/Th2 cytokines [9] and changes in production of other cytokines such as TNF-α and IL-17 [16], [17]. In this regard, we saw a significant increase in CD4+ T cells producing TNF-α alone and also a significant decrease in cells producing IL-2 alone, which may contribute to disease progression despite the increase in the proportion of polyfunctional cells. Indeed, the protective effects of TNF-α have been shown to be dose dependent with too high levels associated with increased TB pathology [16]. A final factor is the immuno-evasion strategies (such as deregulation of macrophage activity) employed by the *Mycobacteria* to inhibit the ability of the immune system to protect against disease progression [18], [19].

Few studies have assessed the contribution of degranulation in the immune response to TB antigens. We wanted to analyse this factor in our study as it is mainly produced by CD8+ T cells, which have been shown to preferentially recognise heavily infected cells *in vitro* thus suggesting a role for them in more advanced stages of disease [3]. Indeed, a recent study has shown distinct differences in CD4+ and CD8+ cytotoxic responses to TB10.4 in a mouse model of TB infection [12]: similar levels were seen early in infection with the CD4+ response decreasing at later stages and the CD8+ response increasing. With loss of CD4+ T cells in HIV-1 infection, a reliance on CD8+ T cells is required but we have recently found this reliance is mono-functional with protection against development of active TB only occurring with restoration of adequate CD4+ T cell levels following initiation of anti-retroviral treatment [20]. In the present study, we found a significant reduction in CD107a expression on both CD4+ and CD8+ cells following successful TB treatment, with levels comparable to HHC. The pattern of expression with other cytokines was quite similar between the two subsets however CD8+ cells showed a reduced level of CD107a following EC stimulation in TB cases post-treatment compared to pre-treatment and HHC. The main limitations of the present study are the relatively small sample size and the lack of longitudinal data and these factors should be included in future work in order to stratify the mono- and polyfunctional CD4+ and CD8+ T cell responses according to distinct stages of TB disease severity.

In conclusion, this study has shown that the response of T cells to TB-specific antigens is increased and more complex in patients with active TB disease compared to their latently infected HHC and that these responses are contracted following successful TB treatment. This suggests that loss of T cell function is not the initial factor in development of active TB disease and supports the requirement for biomarkers of TB disease to account for the stage of disease and bacterial load.

## Methods

### Ethics statement

This study was conducted according to the principles expressed in the Declaration of Helsinki. Ethical approval was obtained from the Gambia Government/Medical Research Council Joint Ethics Committee. All patients provided written informed consent for the collection of samples and subsequent analysis.

### Participants

This was a cross-sectional study involving 12 treatment-naive patients with active TB, 10 patients after TB treatment and 17 *Mycobacterium*-exposed household contacts (HHC) that were consecutively recruited as part of on-going tuberculosis case contact studies at the MRC (UK) unit in The Gambia. Subjects were considered for inclusion if they were >15 years of age and were HIV-1 sero-negative. All cases and contacts underwent a clinical assessment, including a screen for malaria and inter-current illness. Tuberculin skin tests (TST; 2 tuberculin unites [TU], PPD RT23, SSI, Denmark) were performed on the HHC a skin test induration of ≥10 mm diameter categorised as TST positive. Subjects with active disease were recruited into the study if they had 2 consecutive positive smears and were positive by chest x-ray. Of the 12 confirmed TB cases in this study, 7 had mild disease and 5 had severe disease as determined by x-ray score. Other clinical data such as night sweats, cough duration and weight loss were obtained from the TB cases where possible. TB treatment consisted of 2-months intensive phase with rifampicin, pyrazinamide, isoniazid and ethambutol followed by a 4-month continuation phase with isoniazid and rifampicin only. 9/10 subjects analysed at the follow-up time-point had successfully completed treatment with one patient transferring out of the study area and thus unavailable for sputum confirmation. Following informed consent, 10 mL of heparinised blood was taken from all subjects.

### Flow cytometry

#### PBMC separation

Peripheral blood mononuclear cells (PBMC) were isolated using density gradient centrifugation according to the Leucosep® tube protocol (Greiner Bio-one, USA). After two washes, cells were resuspended in RPMI+10% AB serum (Sigma, USA), supplemented with penicillin/streptomycin and L-glutamine and counted using a hemocytometer and trypan blue exclusion.

#### Overnight antigenic stimulation

2 million PBMC resuspended in 1 mL of complete medium were added to single wells of a 24-well plate (Nunc, Germany). Each subject had five different stimulation set-ups: negative (media alone), positive (PHA; 5 µg/mL), PPD-T (10 µg/mL) and ESAT-6/CFP-10 peptides (EC; 10 µg/mL). In addition, CD107a-FITC (20 µL per well; Ebioscience, UK) was added for detection of degranulation. Brefeldin A (10 µg/mL; Sigma USA) and Monensin (0.7 µL/mL; Becton-Dickinson USA) were added after 2 hours and plates incubated overnight at 37°C, 5% CO_2_.

#### Intracellular cytokine staining

Following overnight stimulation, cells were transferred to tubes for flow cytometric acquisition (Dako, USA). Following centrifugation (600_gmax_), supernatant was carefully removed and 20 µL of previously titrated surface marker cocktail was added (CD4-APC-Cy7, CD8-PerCP; BDPharmingen). In addition a live-dead fixable stain was included to enable gating out of any non-viable cells (Invitrogen, USA) Tubes were vortexed and incubated for 15 min. at 4°C. Following washing, 150 µL of Fix/Lyse solution (BDPharmingen) was added per tube, vortexed and incubated at 4°C for 15 min. Cells were washed again and 500 µL of 1× Perm/wash solution (BDpharmingen) was then added; tubes vortexed and incubated for 20 min. at RT in the dark. Following centrifugation the supernatant was carefully removed and 20 µl of cytokine cocktail added (IFN-γ-APC, TNF-α-PECy7 and IL-2-PE; BDPharmingen). Tubes were again vortexed and incubated for 30 min. at RT in the dark, then washed, and cells resuspended in 1% paraformaldehyde (PFA) prior to acquisition.

#### Flow cytometry acquisition and analysis

At least 200,000 lymphocytes were acquired with a CyAn ADP™ (Beckman Coulter, USA) flow cytometer following gating according to 90° forward and side scatter plots. FACS plots were analysed using FlowJo software (version 6.1.1; Treestar, OR). Combinatorial cytokine data were analysed with PESTLE (version 1.5.4) and SPICE (version 4.1.5) software obtained from M. Roederer (National Institutes of Health, Bethesda, MD). Percent frequencies of the different combinations of CD107a, IL-2, TNF-α and IFN-γ+ cells following antigenic stimulation were calculated within the total population of CD4+ or CD8+ T cells and background subtracted (as determined from the medium alone control). Non-specific background was extremely low when more than one cytokine was examined. A cut-off of 0.01% was used as described previously [21]; values below this were set to zero.

### Statistical analysis

Group medians and distributions for TB cases pre- and post-treatment and household contacts (HHC) were compared using the Kruskal-Wallis ANOVA with Dunn's post-test comparison using GraphPad Prism software version 5 (Software MacKiev). Comparison of cytokine combinations was assessed using ANOVA and pie graphs analysed using an in-built permutation test (SPICE version 4.1.5).
